# Temporal trends in the utilization of vasopressors in intensive care units: an epidemiologic study

**DOI:** 10.1186/s40360-016-0063-z

**Published:** 2016-05-07

**Authors:** Charat Thongprayoon, Wisit Cheungpasitporn, Andrew M. Harrison, Perliveh Carrera, Narat Srivali, Wonngarm Kittamongkolchai, Aysen Erdogan, Kianoush B. Kashani

**Affiliations:** Division of Nephrology and Hypertension, Mayo Clinic, 200 First Street SW, Rochester, MN 55905 USA; Medical Scientist Training Program, Mayo Clinic, Rochester, MN USA; Division of Pulmonary and Critical Care Medicine, Mayo Clinic, Rochester, MN USA

**Keywords:** Intensive care unit, Shock, Vasopressors, Epinephrine, Norepinephrine, Phenylephrine, Dopamine, Vasopressin

## Abstract

**Background:**

The choice of vasopressor use in the intensive care unit (ICU) depends primarily on provider preference. This study aims to describe the rate of vasopressor utilization and the trends of each vasoactive agent usage in the ICU over the span of 7 years in a tertiary referral center.

**Methods:**

All adult ICU admissions, including medical, cardiac, and surgical ICUs from January 1st, 2007 through December 31st, 2013 were included in this study. Vasopressor use was defined as the continuous intravenous administration of epinephrine, norepinephrine, phenylephrine, dopamine, or vasopressin within a given ICU day. The vasopressor utilization index (VUI) was defined as the proportion of ICU days on each vasoactive agent divided by the total ICU days with vasopressor usage.

**Results:**

During the study period, 72,005 ICU admissions and 272,271 ICU days were screened. Vasopressors were used in 19,575 ICU admissions (27 %) and 59,811 ICU days (22 %). Vasopressin was used in 24,496 (41 %), epinephrine in 23,229 (39 %), norepinephrine in 20,648 (34 %), dopamine in 9449 (16 %), and phenylephrine in 7508 (13 %) ICU days. The VUI_norepinephrine_ increased from 0.24 in 2007 to 0.46 in 2013 and VUI_phenylephrine_ decreased from 0.20 in 2007 to 0.08 in 2013 (*p* < 0.001 both). For epinephrine, dopamine, and vasopressin VUI did not change over the course of study.

**Conclusion:**

Vasopressors were used in about one fourth of ICU admissions and about one-fifth of ICU days. Although vasopressin is the most commonly used vasopressor, the use of norepinephrine found to have an increasing trajectory.

**Electronic supplementary material:**

The online version of this article (doi:10.1186/s40360-016-0063-z) contains supplementary material, which is available to authorized users.

## Background

Circulatory shock is defined as mismatch among oxygen delivery and tissue consumption which leads to end-organ damage, multisystem organ failure, and potentially death [1, 2]. Patients with circulatory shock commonly present with hypotension. Intravascular fluids and vasoactive agents are often used in the management of these patients. Vasoactive medications which entered clinical practice as of the 1940s are able to increase blood pressure by their vasoconstrictive capabilities.

Between four main types of circulatory shock (hypovolemic, cardiogenic, distributive and obstructive), distributive shock due to sepsis is one the most common forms and is a leading cause of death in non-coronary intensive care units (ICU). Its incidence has been increasing annually [3, 4]. Mortality rate in ICU patients with septic shock ranges from 35 to 60 % [5–7]. In the United States, septic shock is responsible for greater than 200,000 deaths per year [8]. Following optimum fluid resuscitation, treatment with vasoactive agents begins in an effort to restore tissue oxygen delivery and normalize cellular function [9, 10]. The Surviving Sepsis Campaign guidelines for septic shock recommend a mean arterial pressure (MAP) of ≥65 mmHg to achieve adequate end-organ perfusion [11]. Vasoactive agents such as epinephrine, norepinephrine, phenylephrine, dopamine, and vasopressin have increasingly become an integrated therapeutic cornerstone for the management of septic shock. Following the publication of the early goal-directed therapy (EGDT) paper in 2001 [12], there have been a large number of clinical trials with a focus on the efficacy and adverse effects of vasoactive agents for the management of shock [13–23]. Despite the widespread use of vasoactive agents, there are only small numbers of randomized clinical trials to compare their efficacy. In a recent systematic analysis, authors were able to find 23 controlled trials focused on vasopressors [24]. Investigators concluded there was no evidence to support differences among the examined vasopressors. On the other hand, guidelines for the management of circulatory shock have emphasized the use of some of the vasopressors in certain conditions based on their physiology, animal data, and expert opinions.

It is critical to understand the rate of vasopressor utilization and the temporal trend of vasopressor use for the management of shock. This would allow the scientific community to have access to a broader view of current practices and changes over the course of recent years. Thus, we conducted a study to describe the temporal changes in the utilization of vasoactive agents in the ICU setting.

## Methods

### Study population and setting

This is a descriptive study investigating the use of various vasopressors in the ICU setting at a tertiary referral hospital system. All adult ICU admissions of >18 years old from January 1st, 2007 through December 31st, 2013 were examined at Mayo Clinic Hospital in Rochester, MN. Patients without research authorization were excluded. The Mayo Clinic Institutional Board Review approved this study (#14-002385) and waived the consent for patients who had a research authorization.

The Mayo Clinic Rochester Hospital system consists of the Rochester Methodist (342 inpatient beds) and Saint Marys (946 inpatient beds) campuses. Because of the geographic distance between Mayo Clinic and the nearest non-Mayo Clinic ICU, critical care services to the local population are provided exclusively by the Mayo Clinic Rochester Hospital system. This system consists of a total of 138 adult closed ICU beds (Additional file 1: Table S1). Critical care specialists from internal medicine, anesthesiology, and/or surgery background manage and co-manage patients in all ICUs. During the study period, there was no enterprise-level protocol or guideline for vasopressor selection implemented in our ICUs. Thus, vasopressor choices were determined by treating physicians, although, multidisciplinary care provided in our ICUs partly constrains individualized choices for vasoactive agents.

### Data collection

Clinical characteristics and the use of vasopressors was collected using automated retrieval from the institutional electronic medical record system. The use of vasopressors within a given ICU day (12:00 am to 11:59 pm) throughout ICU stay was reviewed. Vasopressor utilization was defined as the continuous intravenous infusion of epinephrine, norepinephrine, phenylephrine, dopamine, or vasopressin, regardless of dosage. Low-dose dopamine was defined as continuous intravenous administration of dopamine at a rate of <3 mcg/kg/min. The number of ICU days on any vasopressor (vasopressor day) and the number of ICU days on each vasopressor were collected. The use of each vasopressor was reported as the vasopressor utilization index (VUI), using the following formula:

$$ \mathrm{Vasopressor}\ \mathrm{utilization}\ \mathrm{index}\ \left(\mathrm{V}\mathrm{U}\mathrm{I}\right)=\frac{\mathrm{The}\ \mathrm{total}\ \mathrm{number}\ \mathrm{of}\ \mathrm{I}\mathrm{C}\mathrm{U}\ \mathrm{days}\ \mathrm{on}\ \mathrm{a}\ \mathrm{given}\ \mathrm{vasopressor}}{\mathrm{The}\ \mathrm{total}\ \mathrm{number}\ \mathrm{of}\ \mathrm{I}\mathrm{C}\mathrm{U}\ \mathrm{days}\ \mathrm{on}\ \mathrm{a}\mathrm{ny}\ \mathrm{vasopressor}} $$

An electronic data extraction algorithm was developed to search for the use of vasopressors within a given ICU day using data from a custom relational research database, which contains a near real-time copy of clinical data from the electronic medical record [25]. This database stores pertinent fluid input/output and Medication Administration Record data within an average of 15 min from entry into the medical record and serves as the data repository for data rules development.

To validate the accuracy of the electronic data extraction algorithm, 300 ICU patients were randomly selected, and comprehensive medical record review was performed for the use of vasopressors within a given ICU day. The algorithm has 97 % sensitivity and 100 % specificity, resulting in a positive predictive value of 100 % and a negative predictive value of 99.6 % (Additional file 1: Table S2).

### Statistical analysis

Continuous variables were reported as mean ± standard deviation (SD) for normally distributed data and median with interquartile range (IQR) for skewed data. Categorical variables were reported as counts and percentages. We calculated VUI for each vasopressor (i.e. epinephrine, norepinephrine, phenylephrine, dopamine, and vasopressin). The trend in the use of each vasopressor in each year from 2007 through 2013 was graphically represented. The annual changes in the rates of VUI were modeled using negative binomial regression (MASS package, Venables, and Ripley, 2002). An offset was utilized in the models to account for the differences in total days of vasopressor use. The analysis was performed in all ICUs and subgroup analysis in each ICU type (medical, surgical, cardiac surgical, cardiac care units, and mixed). *P*-values of < .05 were considered statistically significant. All analyses were performed using JMP statistical software (version 10, SAS, Cary, NC) and R (version 3.1.1; Vienna, Austria).

## Results

From 2007 to 2013, we identified 72,005 ICU admissions and 272,271 ICU days. The median ICU length of stay was 2 (IQR 2–4) days. ICU and in-hospital mortality were 4 and 7 %, respectively. There were no clinically significant differences in characteristics or outcomes among patients admitted to the ICU over the study period (Table 1).

**Table 1:** Clinical characteristics

Characteristics	Total	Year						
		2007	2008	2009	2010	2011	2012	2013
*N*	72,005	10070	10373	10006	10265	10682	10473	10136
Age, year (mean ± SD)	63 ± 17	63 ± 17	63 ± 17	63 ± 17	63 ± 17	63 ± 17	63 ± 17	63 ± 17
Male sex, *n* (%)	41,633 (58)	5741 (57)	5953 (57)	5889 (59)	5928 (58)	6205 (58)	6004 (57)	5913 (58)
White, *n* (%)	66,007 (92)	9117 (91)	9356 (90)	8959 (90)	9452 (92)	9969 (93)	9739 (93)	9415 (93)
BMI, kg/m^2^ (mean ± SD)	29.8 ± 8.0	29.6 ± 7.8	29.6 ± 8.0	29.8 ± 8.1	29.8 ± 8.0	29.8 ± 7.9	29.9 ± 7.9	30.0 ± 8.1
ICU type (%)								
- Cardiac surgery ICU	15,631 (22)	2055 (20)	2104 (20)	2220 (22)	2415 (24)	2257 (21)	2246 (21)	2334 (23)
- Cardiac care unit	8,807 (12)	1372 (14)	1327 (13)	1247 (12)	1206 (12)	1310 (12)	1162 (11)	1183 (12)
- Medical ICU	16,863 (23)	2202 (22)	2315 (22)	2323 (23)	2431 (24)	2527 (24)	2540 (24)	2525 (25)
- Surgical ICU	19,997 (28)	2825 (28)	3046 (29)	2773 (28)	2893 (28)	2908 (27)	2921 (28)	2631 (26)
- Mixed ICU	10,707 (15)	1616 (16)	1581 (15)	1443 (14)	1320 (13)	1680 (16)	1604 (15)	1463 (14)
Admission SOFA score (median, IQR)	4 (2–7)	4 (2–7)	4 (2–7)	4 (2–7)	4 (2–7)	4 (2–7)	4 (2–7)	4 (2–7)
Admission APACHE III score (mean ± SD)	67 ± 26	66 ± 27	67 ± 26	67 ± 26	67 ± 25	68 ± 26	67 ± 26	65 ± 25
Mechanical ventilator use in ICU, *n* (%)	28,844 (40)	4061 (40)	4214 (41)	4183 (42)	4237 (41)	4138 (39)	3904 (37)	4107 (41)
ICU length of stay, day (median, IQR)	2 (2–4)							
ICU mortality, *n* (%)	2,755 (4)	426 (4)	447 (4)	377 (4)	353 (3)	419 (4)	360 (3)	373 (4)
In-hospital mortality, *n* (%)	4,896 (7)	730 (7)	816 (8)	646 (6)	615 (6)	752 (7)	664 (6)	673 (7)

*BMI* body mass index, *SOFA score* the sequential organ failure assessment score, *APACHE III score* the acute physiology and chronic health evaluation III score

Demographic and baseline characteristics of all patients admitted to all ICUs from January 1st, 2007 to December 31st, 2013

Vasopressors were used in 19,575 of ICU admissions (27 %) and 59,811 ICU days (22 %). Vasopressors were most commonly employed in the cardiac surgery ICU (55 % of ICU admissions and 47 % of total ICU days). The vasopressor utilization rate in other ICUs was from 17 to 24 % of ICU admissions and 12 to 23 % of ICU days (Table 2).

**Table 2:** Proportion of ICU patients with vasopressor use divided by total ICU admission and proportion of total ICU day on vasopressor over total ICU day

	ICU admission (*n*)	Patients on vasopressor, *n* (%)	ICU day (*n*)	Vasopressor day, *n* (%)
All ICU	72,005	19,575 (27)	272,271	59,811 (22)
Cardiac surgery ICU	15,631	8,601 (55)	54,212	25,710 (47)
Cardiac care unit	8,807	2,095 (24)	39,732	9,310 (23)
Medical ICU	16,863	2,864 (17)	57,782	8,397 (15)
Surgical ICU	19,997	3,846 (19)	85,065	10,533 (12)
Mixed ICU	10,707	2,169 (20)	35,480	5,861 (17)

Proportion of ICU on vasopressors and vasopressor days

Patients on Vasopressor (%) = Proportion of ICU on with vasopressors = (Total number of ICU on vasopressor/total number of ICU admissions) X100

Vasopressor day (%) = Proportion of ICU vasopressor days = (Total vasopressor day/total ICU day) X100

### Vasopressor utilization in all ICUs

**Fig. 1:**
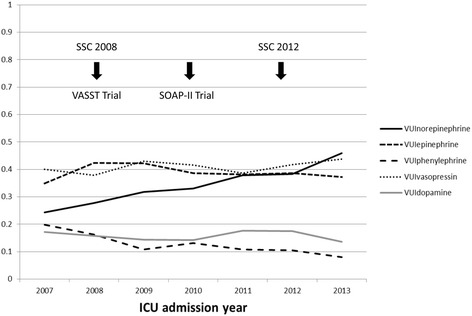
Trend of specific vasopressor use in all ICUs from 2007 to 2013. Note: SSC = Surviving Sepsis Campaign

**Fig. 2:**
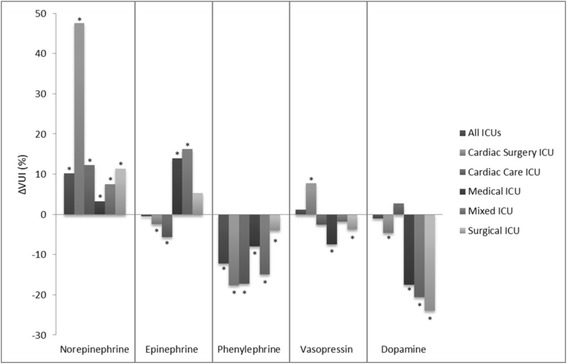
Relative changes in each VUI over the course of study based on ICU type. Note: * = statistically significant

Out of 59,811 vasopressor days, vasopressin was used in 24,496 (41 %), epinephrine in 23,229 (39 %), norepinephrine in 20,648 (34 %), dopamine in 9449 (16 %) and phenylephrine in 7508 (13 %) days. From 2007 to 2013, there was an increasing trend in the use of norepinephrine (VUI_norepinephrine_ was 0.24 in 2007 and 0.46 in 2013; *p* < 0.001) and a decreasing trend in the use of phenylephrine (VUI_phenylephrine_ was 0.20 in 2007 and 0.08 in 2013; *p* < 0.001). Epinephrine, dopamine, and vasopressin utilization trends did not change (Table 3, Additional file 1: Table S3, Figs. 1 and 2). During this period, significant studies and guidelines with the potential effect on VUI were published (Fig. 1).

**Table 3:** Vasopressor Utilization Index with specific vasopressor use from 2007 to 2013

Vasopressor	Average VUI	ICU admission year							ΔVUI (%)	*p*
		2007	2008	2009	2010	2011	2012	2013		
VUI_norepinephrine_	0.34	0.24	0.28	0.32	0.33	0.38	0.38	0.46	+10.3	<0.001
VUI_epinephrine_	0.39	0.35	0.42	0.42	0.39	0.38	0.39	0.37	−0.4	0.73
VUI_phenylephrine_	0.13	0.20	0.16	0.11	0.13	0.11	0.10	0.08	−1.0	<0.001
VUI_vasopressin_	0.41	0.40	0.38	0.43	0.42	0.39	0.42	0.44	+1.3	0.13
VUI_dopamine_	0.16	0.17	0.16	0.14	0.14	0.18	0.17	0.14	−12.2	0.59

VUInorepinephrine = total ICU day on norepinephrine/total ICU day on vasopressor

VUIepinephrine = total ICU day on epinephrine/ total ICU day on vasopressor

VUIphenylephrine = total ICU day on phenylephrine/ total ICU day on vasopressor

VUIvasopressin = total ICU day on vasopressin/total ICU day on vasopressor

VUIdopamine = total ICU day on dopamine/ total ICU day on vasopressor

### Vasopressor use in the cardiac surgery ICU

**Fig. 3:**
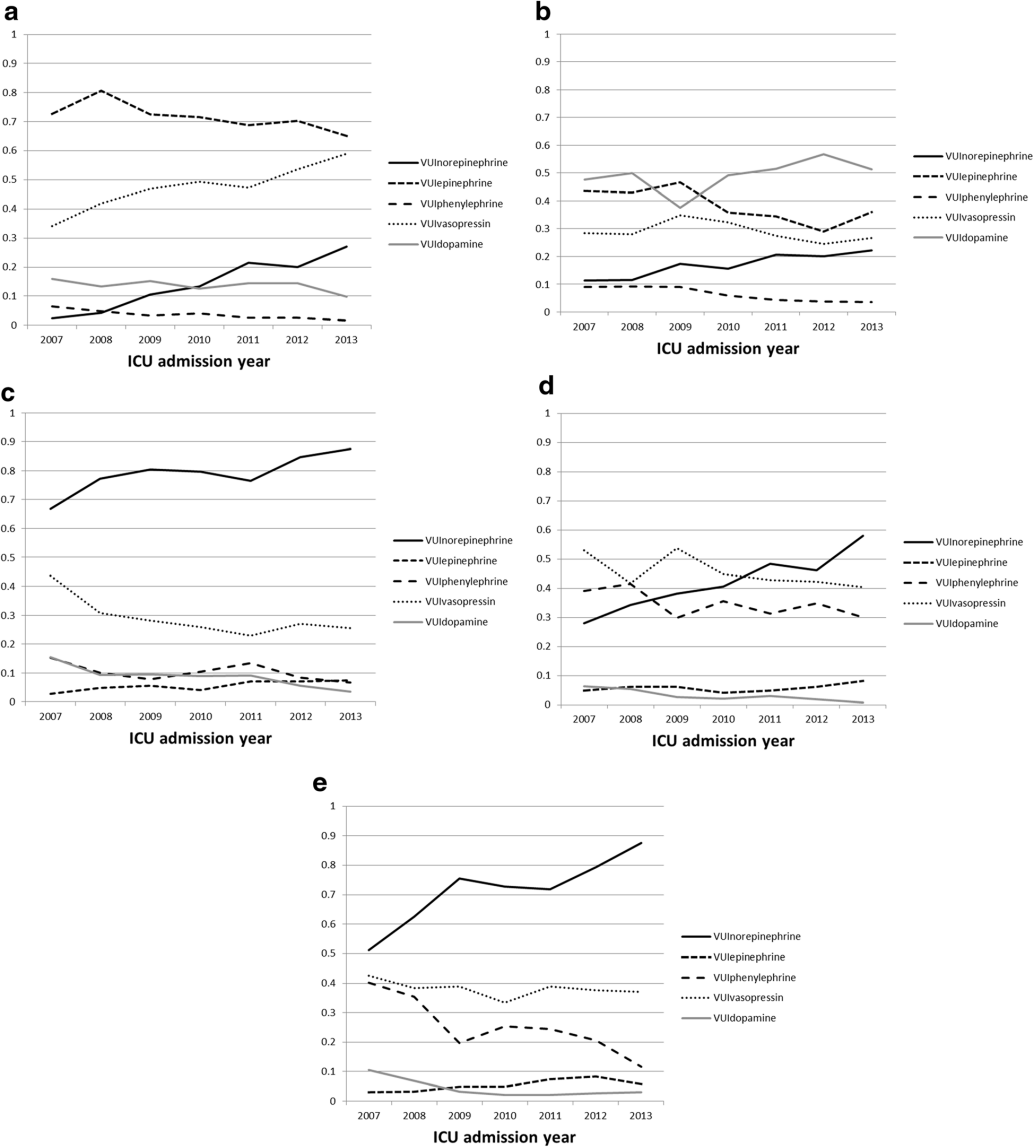
**a** Trend of specific vasopressor use in Cardiac Surgery ICU from 2007 to 2013. **b** Trend of specific vasopressor use in Cardiac Care Unit from 2007 to 2013. **c** Trend of specific vasopressor use in Medical ICU from 2007 to 2013. **d** Trend of specific vasopressor use in Surgical ICU from 2007 to 2013. **e** Trend of specific vasopressor use in Mixed ICU from 2007 to 2013

Out of 25,710 vasopressor days, epinephrine was used in 18,354 (71 %), vasopressin in 12,367 (48 %), norepinephrine in 3838 (15 %), dopamine in 3494 (14 %), and phenylephrine in 905 (4 %) days. From 2007 to 2013, there was an increasing trend in the use of norepinephrine (VUI_norepinephrine_ was 0.02 in 2007 and 0.27 in 2013; *p* < 0.001) and vasopressin (VUI_vasopressin_ was 0.34 in 2007 and 0.59 in 2013; *p* < 0.001). There was a decreasing trend in the use of epinephrine (VUI_epinephrine_ was 0.73 in 2007 and 0.65 in 1013; *p* = 0.001), phenylephrine (VUI_phenylephrine_ was 0.07 in the year 2007 and 0.02 in the year 2013; *p* < 0.001), and dopamine (VUI_dopamine_ was 0.16 in 2007 and 0.10 in 2013; *p* = 0.03) (Additional file 1: Table S4A and Fig. 3a).

### Vasopressor use in the cardiac care unit

Out of 9310 vasopressor days, dopamine was used in 4623 (50 %), epinephrine in 3495 (38 %), vasopressin in 2662 (29 %), norepinephrine in 1638 (18 %), and phenylephrine in 565 (6 %) days. From 2007 to 2013, there was an increasing trend in the use of norepinephrine (VUI_norepinephrine_ was 0.11 in 2007 and 0.22 in 2013; *p* < 0.001). There was a decreasing trend in the use of epinephrine (VUI_epinephrine_ was 0.44 in 2007 and 0.36 in 2013; *p* = 0.002) and phenylephrine (VUI_phenylephrine_ was 0.09 in 2007 and 0.04 in 2013; *p* < 0.001). Vasopressin and dopamine utilization trends did not change (Additional file 1: Table S4B and Fig. 3b).

### Vasopressor use in the medical ICU

Out of 8397 vasopressor days, norepinephrine was used in 6651 (79 %), vasopressin in 2409 (29 %), phenylephrine in 860 (10 %), dopamine in 716 (9 %), and epinephrine in 473 (6 %) days. From 2007 to 2013, there was an increasing trend in the use of norepinephrine (VUI_norepinephrine_ was 0.67 in 2007 and 0.87 in 2013; *p* < 0.001) and epinephrine (VUI_epinephrine_ was 0.03 in 2007 and 0.07 in 2013; *p* < 0.001). There was a decreasing trend in the use of phenylephrine (VUI_phenylephrine_ was 0.15 in 2007 and 0.07 in 2013; *p* = 0.04), vasopressin (VUI_vasopressin_ was 0.44 in 2007 and 0.26 in 2013; *p* = 0.001), and dopamine (VUI_dopamine_ was 0.15 in 2007 and 0.04 in 2013; *p* < 0.001), (Additional file 1: Table S4C and Fig. 3c).

### Vasopressor use in the surgical ICU

Out of 10,533 vasopressor days, vasopressin was used in 4817 (46 %), norepinephrine in 4357 (41 %), phenylephrine in 3673 (35 %), epinephrine in 599 (6 %) and dopamine in 349 (3 %) days. From 2007 to 2013, there was an increasing trend in the use of norepinephrine (VUI_norepinephrine_ was 0.28 in 2007 and 0.58 in 2013; *p* < 0.001). There was a decreasing trend in the use of phenylephrine (VUI_phenylephrine_ was 0.39 in 2007 and 0.30 in 2013; *p* = 0.02), vasopressin (VUI_vasopressin_ was 0.53 in 2007 and 0.40 in 2013; *p* = 0.01) and dopamine (VUI_dopamine_ was 0.06 in 2007 and 0.01 in 2013; *p* < 0.001). Epinephrine utilization trend did not change (Additional file 1: Table S4D and Fig. 3d).

### Vasopressor use in the mixed medical/surgical ICU

Out of 5861 vasopressor days, norepinephrine was used in 4164 (71 %), vasopressin in 2241 (38 %), phenylephrine in 1505 (26 %), epinephrine in 308 (5 %), and dopamine in 267 (5 %) days. From 2007 to 2013, there was an increasing trend of norepinephrine (VUI_norepinephrine_ was 0.51 in 2007 and 0.88 in 2013; *p* < 0.001) and epinephrine (VUI_epinephrine_ was 0.03 in the year 2007 and 0.06 in the year 2013; *p* < 0.001). There was a decreasing trend in the use of phenylephrine (VUI_phenylephrine_ was 0.40 in 2007 and 0.12 in 2013; *p* < 0.001) and dopamine (VUI_dopamine_ was 0.10 in 2007 and 0.03 in 2013; *p* = 0.001). Vasopressin utilization trends did not change (Additional file 1: Table S4E and Fig. 3e).

### Low-dose dopamine use in the ICU

**Fig. 4:**
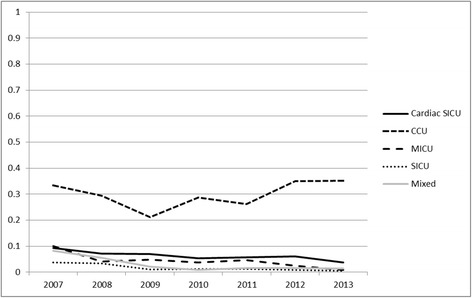
Trend of the use of low-dose dopamine (<3 mcg/kg/min) in ICU subgroups

In the cardiac care unit, use of low-dose dopamine is still common (VUI_low-dose dopamine_ was 0.30) without any decreasing trend in its utilization. In comparison, the use of low-dose dopamine is much less frequent with a downward trend in other ICUs over this 7 year period. In 2013, VUI_low-dose dopamine_ was 0.01 in the medical, surgical, and mixed ICUs and 0.06 in cardiac surgery ICU (Additional file 1: Table S5 and Fig. 4).

## Discussion

Vasoactive agents were used in about one-fourth of ICU admissions and ICU days. Overall, vasopressin was the most commonly used vasopressor from 2007 to 2013, following by epinephrine, norepinephrine, dopamine, and phenylephrine. The use of norepinephrine showed a significant increasing trend toward becoming the most commonly used vasopressor in all ICUs in 2013.

An increasing trend in the use of norepinephrine in all ICUs is not surprising. Norepinephrine has been recommended as the first choice among vasoactive agents by the Surviving Sepsis Campaign since 2004 [11, 26, 27]. The increasing awareness and adoption of Surviving Sepsis Campaign are likely to have an impact on the choice of vasopressor utilization in ICU [28, 29]. In 2010, a multicenter randomized trial subgroup analysis demonstrated that patients with cardiogenic shock who received dopamine had a higher mortality compared to norepinephrine [14]. These two examples offer significant insight into the rise in the rate of the norepinephrine use after 2010.

Following the SOAP-II trial [14], De Backer and colleagues conducted a meta-analysis [15] to compare the use of dopamine versus norepinephrine in the treatment of septic shock. They demonstrated that dopamine administration was associated with greater mortality and a higher incidence of arrhythmia compared to norepinephrine. Dopamine not only provides different effects at varying dosages [30, 31], but each individual hemodynamic responses is different to this vasoactive agent [32, 33]. For this reason, the Surviving Sepsis Campaign suggests dopamine not to be used as an alternative to norepinephrine in septic shock [11]. In addition, low-dose dopamine is recommended not to be used as a renal-protective strategy. Our study demonstrated a significant downward trend in the dopamine use in medical and mixed ICUs where the septic shock was expected to be the most common reason for vasopressor use. Interestingly, we found that the use of dopamine is only minimally reduced in all ICUs. Despite a decreasing trend in the use of dopamine in medical, surgical, cardiac surgery and mixed ICUs, our study shows dopamine is still the most commonly used vasopressor agent in the cardiac care unit.

Approximately one-third of patients with septic shock are vasopressin deficient [34, 35]. Although vasopressin, especially in high doses, has been demonstrated to be very effective, concerns exist regarding its adverse effects on peripheral, intestinal, and renal circulation [36–38]. Vasopressin was the most commonly used vasopressor from 2007 to 2013 in all ICUs. This was mainly due to an increasing trend in vasopressin use in Cardiac surgery ICU. In contrast, there was a decreasing trend in vasopressin use in Medical and Surgical ICU. The VASST trial showed in patients with septic shock, low-dose vasopressin did not decrease mortality rates when compared to norepinephrine [13]. Given being more costly with unclear benefit, vasopressin might lose its favor for use in septic shock. The Surviving Sepsis Campaign recommended against the use of vasopressin as a single initial vasopressor for septic shock [11].

The future use of vasopressors in ICUs may change as a result of recent randomized controlled trials conducted by ProCESS investigators [39] from the United States and ARISE investigators from Australia & New Zealand [40, 41]. Early Goal Directed Therapy (EGDT) and the outcomes of patients with septic shock were studied in all three trials. All studies found that EGDT did not reduce all-cause mortality in critically ill patients with early septic shock. In addition, titrating therapy to central venous pressure or central oxygen saturation was not more effective than usual care in patients with severe sepsis [42]. These recent findings indicate that modification to the previously accepted EGDT algorithm may result in as much as a 10–15 % reduction in the overall use of vasopressor for severe sepsis and septic shock. The SEPSISPAM investigators [18] recently conducted a multicenter trial in 776 patients with septic shock to undergo resuscitation with a high-target MAP of 80 to 85 mmHg or low-target MAP of 65 to 70 mmHg. Although no clear benefit to higher MAP targets was found in the general population with septic shock, this study showed a decrease in the incidence of acute kidney injury and the need for renal replacement therapy in the patients with chronic hypertension when they were assigned to higher MAP arm. If future studies confirm this finding, it has the potential to change the rate of vasopressor utilization in the ICUs.

There are many potential explanations as to the cause of the changes in vasopressor utilization observed in this study. These explanations include temporal changes in standard practices of care, staffing changes, staffing preferences, and patient characteristics. Stratification by specific patient characteristics, such as primary diagnosis (septic shock, cardiogenic shock), and comorbid diagnoses may further explain these differences. Temporal changes in stratification of specific patient characteristics may be present as well. However, the cause of the observations of this study ultimately remains unknown. Thus, further study is required to understand the contribution of each of these potential causal relationships to these observations.

This study has several limitations. 1) This is a single-center, retrospective study. This limits the generalizability of some results, especially to the non-academic medical setting. In addition, the majority of included patients were post-cardiac surgery patients. 2) Causes or types of shock, as well as patient comorbidities, may affect the choice of vasopressors used. Although these factors may partly explain variations in vasopressor use, we did not investigate the use of vasopressor in each type of shock because the determination of shock type was not trivial, particularly in a retrospective study. For example, type of shock was often unknown, or multiple types of shock occurred simultaneously. As the severity of illness was not changed during the course of study, we believe changes in the use of vasopressors are mainly due to the growing knowledge of the nature of each vasopressor in critical illness.

On the other hand, our study carries several strengths. To our knowledge, this is the first study to report the temporal trend in utilization of each vasopressor in a cohort of ICU patients. As our center covers a very broad referral base, the diversity of patients and their underlying pathologies provided appropriate depth to our findings. Multidisciplinary care provided in our ICUs limits individualized choices for vasoactive agents. Our sample size is rather large and this allows appropriate subgroup analyses on the basis of ICU type that provide additional insight of vasopressor utilization in each specific ICU type.

## Conclusions

Utilization of vasoactive medications has changed over the past decade. This could be due to the growth of the knowledge regarding the performance of each vasoactive agent. Although we found vasopressin was the most commonly used vasopressor from 2007 to 2013, norepinephrine has taken its place as the most commonly used vasopressor in the ICUs, as of 2013. We also noted dopamine is still used very often in the cardiac care unit. Our study provides a large perspective view of vasoactive agent utilization which could be utilized in the future trials to improve the choices if these agents based on each patients population.

### Ethics

The Mayo Clinic Institutional Board Review approved this study (#14-002385) and waived the consent for patients who had a research authorization.

### Consent to publish

Not applicable.

## Availability of data and materials

All data supporting the findings is contained within the manuscript.

## Additional file

Additional file 1:
**Table S1.** ICU types and patient volumes in 2012 and 2013. **Table S2.** Validation of electronic data extraction for vasopressors. **Table S3.** Relative changes in each vasopressor VUI over the course of study based on ICU type. **Table S4.** (A through E): Vasopressor Utilization Index with specific vasopressor use in all ICU subgroups from 2007 through 2013. **Table S5.** Trend of the use of low-dose dopamine (<3 mcg/kg/min) in ICUs and ICU subgroups. (DOCX 23 kb)

## Abbreviations

EGDT: early goal-directed therapy
ICU: intensive care unit
IQR: interquartile range
MAP: mean arterial pressure
SD: standard deviation
VUI: vasopressor utilization index

## References

[1] Bonanno FG (2011). Clinical pathology of the shock syndromes. J Emerg Trauma Shock.

[2] Bonanno FG (2012). Shock - A reappraisal: The holistic approach. J Emerg Trauma Shock.

[3] Mayr FB, Yende S, Angus DC (2014). Epidemiology of severe sepsis. Virulence.

[4] Nasa P, Juneja D, Singh O (2012). Severe sepsis and septic shock in the elderly: An overview. World J Crit Care Med.

[5] Bone RC (1992). Toward an epidemiology and natural history of SIRS (systemic inflammatory response syndrome). JAMA.

[6] Beal AL, Cerra FB (1994). Multiple organ failure syndrome in the 1990s. Systemic inflammatory response and organ dysfunction. JAMA.

[7] Fourrier F, Chopin C, Goudemand J, Hendrycx S, Caron C, Rime A, Marey A, Lestavel P (1992). Septic shock, multiple organ failure, and disseminated intravascular coagulation. Compared patterns of antithrombin III, protein C, and protein S deficiencies. Chest.

[8] Angus DC, Linde-Zwirble WT, Lidicker J, Clermont G, Carcillo J, Pinsky MR (2001). Epidemiology of severe sepsis in the United States: analysis of incidence, outcome, and associated costs of care. Crit Care Med.

[9] Hollenberg SM (2007). Vasopressor support in septic shock. Chest.

[10] Hollenberg SM, Ahrens TS, Annane D, Astiz ME, Chalfin DB, Dasta JF, Heard SO, Martin C, Napolitano LM, Susla GM, Totaro R, Vincent J-L, Zanotti-Cavazzoni S (2004). Practice parameters for hemodynamic support of sepsis in adult patients: 2004 update. Crit Care Med.

[11] Dellinger RP, Levy MM, Rhodes A, Annane D, Gerlach H, Opal SM, Sevransky JE, Sprung CL, Douglas IS, Jaeschke R, Osborn TM, Nunnally ME, Townsend SR, Reinhart K, Kleinpell RM, Angus DC, Deutschman CS, Machado FR, Rubenfeld GD, Webb SA, Beale RJ, Vincent JL, Moreno R (2013). Surviving sepsis campaign: international guidelines for management of severe sepsis and septic shock: 2012. Crit Care Med.

[12] Rivers E, Nguyen B, Havstad S, Ressler J, Muzzin A, Knoblich B, Peterson E, Tomlanovich M (2001). Early goal-directed therapy in the treatment of severe sepsis and septic shock. N Engl J Med.

[13] Russell JA, Walley KR, Singer J, Gordon AC, Hebert PC, Cooper DJ, Holmes CL, Mehta S, Granton JT, Storms MM, Cook DJ, Presneill JJ, Ayers D (2008). Vasopressin versus norepinephrine infusion in patients with septic shock. N Engl J Med.

[14] De Backer D, Biston P, Devriendt J, Madl C, Chochrad D, Aldecoa C, Brasseur A, Defrance P, Gottignies P, Vincent JL (2010). Comparison of dopamine and norepinephrine in the treatment of shock. N Engl J Med.

[15] De Backer D, Aldecoa C, Njimi H, Vincent JL (2012). Dopamine versus norepinephrine in the treatment of septic shock: a meta-analysis*. Crit Care Med.

[16] Lampard JG, Lang E (2013). Vasopressors for hypotensive shock. Ann Emerg Med.

[17] Morelli A, Ertmer C, Westphal M, Rehberg S, Kampmeier T, Ligges S, Orecchioni A, D’Egidio A, D’Ippoliti F, Raffone C, Venditti M, Guarracino F, Girardis M, Tritapepe L, Pietropaoli P, Mebazaa A, Singer M (2013). Effect of heart rate control with esmolol on hemodynamic and clinical outcomes in patients with septic shock: a randomized clinical trial. JAMA.

[18] Asfar P, Meziani F, Hamel JF, Grelon F, Megarbane B, Anguel N, Mira JP, Dequin PF, Gergaud S, Weiss N, Legay F, Le Tulzo Y, Conrad M, Robert R, Gonzalez F, Guitton C, Tamion F, Tonnelier JM, Guezennec P, Van Der Linden T, Vieillard-Baron A, Mariotte E, Pradel G, Lesieur O, Ricard JD, Herve F, du Cheyron D, Guerin C, Mercat A, Teboul JL, Radermacher P (2014). High versus low blood-pressure target in patients with septic shock. N Engl J Med.

[19] Chawla LS, Busse L, Brasha-Mitchell E, Davison D, Honiq J, Alotaibi Z, Seneff MG (2014). Intravenous angiotensin II for the treatment of high-output shock (ATHOS trial): a pilot study. Crit Care.

[20] Russell JA, Walley KR, Gordon AC, Cooper DJ, Hebert PC, Singer J, Holmes CL, Mehta S, Granton JT, Storms MM, Cook DJ, Presneill JJ (2009). Interaction of vasopressin infusion, corticosteroid treatment, and mortality of septic shock. Crit Care Med.

[21] Lauzier F, Levy B, Lamarre P, Lesur O (2006). Vasopressin or norepinephrine in early hyperdynamic septic shock: a randomized clinical trial. Intensive Care Med.

[22] Myburgh JA, Higgins A, Jovanovska A, Lipman J, Ramakrishnan N, Santamaria J (2008). A comparison of epinephrine and norepinephrine in critically ill patients. Intensive Care Med.

[23] Morelli A, Ertmer C, Rehberg S, Lange M, Orecchioni A, Laderchi A, Bachetoni A, D’Alessandro M, Van Aken H, Pietropaoli P, Westphal M (2008). Phenylephrine versus norepinephrine for initial hemodynamic support of patients with septic shock: a randomized, controlled trial. Crit Care.

[24] Gamper G, Havel C, Arrich J, Losert H, Pace NL, Müllner M, Herkner H (2016). Vasopressors for hypotensive shock. Cochrane Database Syst Rev.

[25] Herasevich V, Pickering BW, Dong Y, Peters SG, Gajic O (2010). Informatics infrastructure for syndrome surveillance, decision support, reporting, and modeling of critical illness. Mayo Clin Proc.

[26] Dellinger RP, Carlet JM, Masur H, Gerlach H, Calandra T, Cohen J, Gea-Banacloche J, Keh D, Marshall JC, Parker MM, Ramsay G, Zimmerman JL, Vincent JL, Levy MM (2004). Surviving Sepsis Campaign guidelines for management of severe sepsis and septic shock. Crit Care Med.

[27] Dellinger RP, Levy MM, Carlet JM, Bion J, Parker MM, Jaeschke R, Reinhart K, Angus DC, Brun-Buisson C, Beale R, Calandra T, Dhainaut JF, Gerlach H, Harvey M, Marini JJ, Marshall J, Ranieri M, Ramsay G, Sevransky J, Thompson BT, Townsend S, Vender JS, Zimmerman JL, Vincent JL (2008). Surviving Sepsis Campaign: international guidelines for management of severe sepsis and septic shock: 2008. Crit Care Med.

[28] Levy MM, Dellinger RP, Townsend SR, Linde-Zwirble WT, Marshall JC, Bion J, Schorr C, Artigas A, Ramsay G, Beale R, Parker MM, Gerlach H, Reinhart K, Silva E, Harvey M, Regan S, Angus DC (2010). The Surviving Sepsis Campaign: results of an international guideline-based performance improvement program targeting severe sepsis. Crit Care Med.

[29] Levy MM, Rhodes A, Phillips GS, Townsend SR, Schorr CA, Beale R, Osborn T, Lemeshow S, Chiche JD, Artigas A, Dellinger RP (2015). Surviving sepsis campaign: association between performance metrics and outcomes in a 7.5-year study. Crit Care Med.

[30] de la Cal MA, Miravalles E, Pascual T, Esteban A, Ruiz-Santana S (1984). Dose-related hemodynamic and renal effects of dopamine in septic shock. Crit Care Med.

[31] Filseth OM, How OJ, Kondratiev T, Gamst TM, Sager G, Tveita T (2012). Changes in cardiovascular effects of dopamine in response to graded hypothermia in vivo. Crit Care Med.

[32] Timmis AD, Fowler MB, Chamberlain DA (1981). Comparison of haemodynamic responses to dopamine and salbutamol in severe cardiogenic shock complicating acute myocardial infarction. Br Med J (Clin Res Ed).

[33] Regnier B, Rapin M, Gory G, Lemaire F, Teisseire B, Harari A (1977). Haemodynamic effects of dopamine in septic shock. Intensive Care Med.

[34] Landry DW, Levin HR, Gallant EM, Ashton RC, Seo S, D’Alessandro D, Oz MC, Oliver JA (1997). Vasopressin deficiency contributes to the vasodilation of septic shock. Circulation.

[35] Sharshar T, Blanchard A, Paillard M, Raphael JC, Gajdos P, Annane D (2003). Circulating vasopressin levels in septic shock. Crit Care Med.

[36] Krejci V, Hiltebrand LB, Jakob SM, Takala J, Sigurdsson GH (2007). Vasopressin in septic shock: effects on pancreatic, renal, and hepatic blood flow. Crit Care.

[37] Kahn JM, Kress JP, Hall JB (2002). Skin necrosis after extravasation of low-dose vasopressin administered for septic shock. Crit Care Med.

[38] Holmes CL, Walley KR (2004). Vasopressin in the ICU. Curr Opin Crit Care.

[39] Yealy DM, Kellum JA, Huang DT, Barnato AE, Weissfeld LA, Pike F, Terndrup T, Wang HE, Hou PC, LoVecchio F, Filbin MR, Shapiro NI, Angus DC (2014). A randomized trial of protocol-based care for early septic shock. N Engl J Med.

[40] Peake SL, Delaney A, Bailey M, Bellomo R, Cameron PA, Cooper DJ, Higgins AM, Holdgate A, Howe BD, Webb SA, Williams P (2014). Goal-directed resuscitation for patients with early septic shock. N Engl J Med.

[41] Mouncey PR, Osborn TM, Power GS, Harrison DA, Sadique MZ, Grieve RD, Jahan R, Harvey SE, Bell D, Bion JF, Coats TJ, Singer M, Young JD, Rowan KM (2015). Trial of early, goal-directed resuscitation for septic shock. N Engl J Med.

[42] Lilly CM (2014). The ProCESS trial--a new era of sepsis management. N Engl J Med.

