# Supplementary material for: Temporal trends in the utilization of vasopressors in intensive care units: an epidemiologic study
Source: BMC Pharmacol Toxicol. 2016 May 7;17:19. doi: 10.1186/s40360-016-0063-z (PMC4859949; doi:10.1186/s40360-016-0063-z)
Supplement: Additional file 1: — Table S1. ICU types and patient volumes in 2012 and 2013. Table S2. Validation of electronic data extraction for vasopressors. Table S3. Relative changes in each vasopressor VUI over the course of study based on ICU type. Table S4. (A through E): Vasopressor Utilization Index with specific vasopressor use in all ICU subgroups from 2007 through 2013. Table S5. Trend of the use of low-dose dopamine (<3 mcg/kg/min) in ICUs and ICU subgroups. (DOCX 23 kb) [file 40360_2016_63_MOESM1_ESM.docx]

**Table S1** ICU types and patient volumes in 2012 and 2013

| ICU type | Unit | Bed | 2012 | | | 2013 | | |
| --- | --- | --- | --- | --- | --- | --- | --- | --- |
|  |  |  | Patient days | admission | Average LOS | Patient days | admission | Average LOS |
| Cardiac surgery | Cardiac Surgery ICU | 25 | 6,245 | 2,243 | 2.8 | 6,990 | 2,353 | 2.9 |
|  | Cardiac Surgery ICU  (heart and lung transplant) | 8 | 1,568 | 461 | 3.2 | 753 | 516 | 1.5 |
| Cardiac | Cardiac Care Unit | 16 | 3,930 | 1,304 | 3 | 4,082 | 1,341 | 2.9 |
| Medical | Medical ICU | 24 | 6,179 | 2,800 | 2.2 | 6,031 | 2,818 | 2.2 |
| Surgical | Trauma and general surgery ICU | 24 | 6,471 | 1,722 | 3.7 | 5,001 | 1,521 | 3.3 |
|  | Vascular and thoracic surgery ICU | 20 | 3,803 | 1,584 | 2.4 | 3,261 | 1,408 | 2.3 |
| Mixed | Medical, Surgical, liver transplant ICU | 21 | 3,813 | 1,713 | 2.2 | 3,589 | 1,570 | 2.2 |
|  | Total | 138 | 32,009 | 11,827 | 3.2 | 29,707 | 11,527 | 3.0 |

LOS, Length of stay.

**Table S2:** Validation of electronic data extraction for vasopressors

|  | Manual Vasopressor  (Yes) | Manual vasopressor  (No) | Total |
| --- | --- | --- | --- |
| Electronic vasopressor (Yes) | 31 | 0 | 31 |
| Electronic vasopressor (No) | 1 | 268 | 269 |
| Total | 32 | 268 | 300 |

Sensitivity 97%, specificity 100%, positive predictive value 100%, negative predictive value 99.6%

**Table S3:** Relative changes in each vasopressor VUI over the course of study based on ICU type.

|  | All ICUs | | Cardiac Surgery ICU | | Cardiac Care ICU | | Medical ICU | | Mixed ICU | | Surgical ICU | |
| --- | --- | --- | --- | --- | --- | --- | --- | --- | --- | --- | --- | --- |
|  | **ΔVUI (%)** | **p-value** | **ΔVUI (%)** | **p-value** | **ΔVUI (%)** | **p-value** | **ΔVUI (%)** | **p-value** | **ΔVUI (%)** | **p-value** | **ΔVUI (%)** | **p-value** |
| **Norepinephrine** | +10.3 | <0.0001 | +47.6 | <0.0001 | +12.3 | <0.001 | +3.3 | <0.001 | +7.5 | <0.001 | +11.4 | <0.001 |
| **Epinephrine** | -0.4 | 0.73 | -2.4 | 0.0006 | -5.7 | 0.002 | +14.0 | <0.001 | +16.3 | <0.001 | +5.3 | 0.14 |
| **Phenylephrine** | -12.2 | <0.0001 | -17.7 | <0.0001 | -17.2 | <0.001 | -7.9 | 0.04 | -14.9 | <0.001 | -3.9 | 0.02 |
| **Vasopressin** | +1.3 | 0.13 | +7.8 | <0.0001 | -2.5 | 0.18 | -7.4 | 0.001 | -1.7 | 0.09 | -3.7 | 0.01 |
| **Dopamine** | -0.1 | 0.59 | -4.6 | 0.03 | +2.8 | 0.15 | -17.4 | <0.001 | -20.5 | <0.001 | -23.9 | <0.001 |

*p-value < 0.05

**Table S4:** VUI and Trend of specific vasopressor use in all ICU subgroups from 2007 through 2013

1. Cardiac surgery ICU

| Vasopressor | Average  VUI | ICU admission year | | | | | | | p |
| --- | --- | --- | --- | --- | --- | --- | --- | --- | --- |
|  |  | 2007 | 2008 | 2009 | 2010 | 2011 | 2012 | 2013 |  |
| VUI_norepinephrine_ | 0.15 | 0.02 | 0.04 | 0.11 | 0.13 | 0.22 | 0.20 | 0.27 | <0.001 |
| VUI_epinephrine_ | 0.71 | 0.73 | 0.80 | 0.73 | 0.72 | 0.69 | 0.70 | 0.65 | 0.001 |
| VUI_phenylephrine_ | 0.04 | 0.07 | 0.05 | 0.03 | 0.04 | 0.03 | 0.03 | 0.02 | <0.001 |
| VUI_vasopressin_ | 0.48 | 0.34 | 0.42 | 0.47 | 0.49 | 0.47 | 0.54 | 0.59 | <0.001 |
| VUI_dopamine_ | 0.14 | 0.16 | 0.13 | 0.15 | 0.13 | 0.14 | 0.14 | 0.10 | 0.03 |

1. Cardiac care unit

| Vasopressor | Average VUI | ICU admission year | | | | | | | p |
| --- | --- | --- | --- | --- | --- | --- | --- | --- | --- |
|  |  | 2007 | 2008 | 2009 | 2010 | 2011 | 2012 | 2013 |  |
| VUI_norepinephrine_ | 0.18 | 0.11 | 0.11 | 0.17 | 0.16 | 0.21 | 0.20 | 0.22 | <0.001 |
| VUI_epinephrine_ | 0.38 | 0.44 | 0.42 | 0.47 | 0.36 | 0.34 | 0.29 | 0.36 | 0.002 |
| VUI_phenylephrine_ | 0.06 | 0.09 | 0.09 | 0.09 | 0.06 | 0.04 | 0.04 | 0.04 | <0.001 |
| VUI_vasopressin_ | 0.29 | 0.28 | 0.28 | 0.35 | 0.32 | 0.28 | 0.25 | 0.27 | 0.18 |
| VUI_dopamine_ | 0.50 | 0.48 | 0.50 | 0.37 | 0.49 | 0.52 | 0.57 | 0.51 | 0.15 |

1. Medical ICU

| Vasopressor | Average VUI | ICU admission year | | | | | | | p |
| --- | --- | --- | --- | --- | --- | --- | --- | --- | --- |
|  |  | 2007 | 2008 | 2009 | 2010 | 2011 | 2012 | 2013 |  |
| VUI_norepinephrine_ | 0.79 | 0.67 | 0.77 | 0.80 | 0.80 | 0.77 | 0.85 | 0.87 | <0.001 |
| VUI_epinephrine_ | 0.06 | 0.03 | 0.05 | 0.06 | 0.04 | 0.07 | 0.07 | 0.07 | <0.001 |
| VUI_phenylephrine_ | 0.10 | 0.15 | 0.10 | 0.08 | 0.10 | 0.13 | 0.08 | 0.07 | 0.04 |
| VUI_vasopressin_ | 0.29 | 0.44 | 0.31 | 0.28 | 0.26 | 0.23 | 0.27 | 0.26 | 0.001 |
| VUI_dopamine_ | 0.09 | 0.15 | 0.09 | 0.09 | 0.09 | 0.09 | 0.06 | 0.04 | <0.001 |

1. Surgical ICU

| Vasopressor | Average VUI | ICU admission year | | | | | | | p |
| --- | --- | --- | --- | --- | --- | --- | --- | --- | --- |
|  |  | 2007 | 2008 | 2009 | 2010 | 2011 | 2012 | 2013 |  |
| VUI_norepinephrine_ | 0.41 | 0.28 | 0.34 | 0.38 | 0.41 | 0.48 | 0.46 | 0.58 | <0.001 |
| VUI_epinephrine_ | 0.06 | 0.05 | 0.06 | 0.06 | 0.04 | 0.05 | 0.06 | 0.08 | 0.14 |
| VUI_phenylephrine_ | 0.35 | 0.39 | 0.42 | 0.30 | 0.36 | 0.31 | 0.35 | 0.30 | 0.02 |
| VUI_vasopressin_ | 0.46 | 0.53 | 0.42 | 0.54 | 0.45 | 0.43 | 0.42 | 0.40 | 0.01 |
| VUI_dopamine_ | 0.03 | 0.06 | 0.05 | 0.03 | 0.02 | 0.03 | 0.02 | 0.01 | <0.001 |

1. Mixed ICU

| Vasopressor | Average VUI | ICU admission year | | | | | | | p |
| --- | --- | --- | --- | --- | --- | --- | --- | --- | --- |
|  |  | 2007 | 2008 | 2009 | 2010 | 2011 | 2012 | 2013 |  |
| VUI_norepinephrine_ | 0.71 | 0.51 | 0.62 | 0.76 | 0.73 | 0.72 | 0.79 | 0.88 | <0.001 |
| VUI_epinephrine_ | 0.05 | 0.03 | 0.03 | 0.05 | 0.05 | 0.07 | 0.08 | 0.06 | <0.001 |
| VUI_phenylephrine_ | 0.26 | 0.40 | 0.35 | 0.20 | 0.25 | 0.24 | 0.21 | 0.12 | <0.001 |
| VUI_vasopressin_ | 0.38 | 0.43 | 0.38 | 0.39 | 0.33 | 0.39 | 0.38 | 0.37 | 0.09 |
| VUI_dopamine_ | 0.05 | 0.10 | 0.07 | 0.03 | 0.02 | 0.02 | 0.02 | 0.03 | 0.001 |

**Table S5:** Trend of the use of low-dose dopamine (< 3 mcg/kg/min) in ICUs and ICU subgroups

| Vasopressor | Average VUI | ICU admission year | | | | | | |
| --- | --- | --- | --- | --- | --- | --- | --- | --- |
|  |  | 2007 | 2008 | 2009 | 2010 | 2011 | 2012 | 2013 |
| All | 0.09 | 0.11 | 0.09 | 0.07 | 0.07 | 0.08 | 0.09 | 0.08 |
| Cardiac SICU | 0.06 | 0.09 | 0.07 | 0.07 | 0.05 | 0.06 | 0.06 | 0.04 |
| Cardiac care unit | 0.30 | 0.33 | 0.29 | 0.21 | 0.29 | 0.26 | 0.35 | 0.35 |
| Medical ICU | 0.04 | 0.10 | 0.04 | 0.05 | 0.04 | 0.05 | 0.01 | 0.01 |
| Surgical ICU | 0.02 | 0.04 | 0.03 | 0.01 | 0.01 | 0.01 | 0.01 | 0.01 |
| Mixed ICU | 0.03 | 0.08 | 0.05 | 0.02 | 0.01 | 0.02 | 0.01 | 0.01 |
